# Correlation between hyperglycemia and glycated albumin with retinopathy of prematurity

**DOI:** 10.1038/s41598-021-01861-8

**Published:** 2021-11-16

**Authors:** Ana C. Almeida, Gabriela A. Silva, Gabriele Santini, Margarida Brízido, Miguel Correia, Constança Coelho, Luís Miguel Borrego

**Affiliations:** 1 Department of Ophthalmology, Hospital Beatriz Angelo, Loures, Av. Carlos Teixeira 3, 2674-514 Loures, Portugal; 2 Neonatal Intensive Care Unit, Hospital São Francisco Xavier – Centro Hospitalar de Lisboa Ocidental, Estr. Forte do Alto Duque, 1449-005 Lisbon, Portugal; 3 CEDOC, Chronic Diseases Research Center, NOVA Medical School – Universidade Nova de Lisboa, Campo dos Mártires da Pátria 130, 1169-056 Lisbon, Portugal; 4 Comprehensive Health Research Centre (CHRC), NOVA Medical School, Universidade Nova de Lisboa, Campo dos Mártires da Pátria, 1169-056 Lisbon, Portugal; 5 Department of Ophthalmology, Luz Saúde, Hospital da Luz, Av. Lusíada 100, 1500-650 Lisbon, Portugal; 6 iNOVA4Health, CEDOC, NOVA Medical School, NMS, Universidade Nova de Lisboa, Campo dos Mártires da Pátria, 1169-056 Lisbon, Portugal; 7 R&D Department, Instrumentation Laboratory - A Werfen Company, Viale Monza, 338, 20128 Milan, Italy; 8 Escola Superior de Saúde Egas Moniz, Campus Universitário, Quinta da Granja, Monte de Caparica, 2829-511 Almada, Portugal; 9 Faculdade Medicina de Lisboa, Institute of Environmental Health (ISAMB), University of Lisbon, Av. Prof. Egas Moniz MB, 1649-028 Lisboa, Portugal; 10 Department of Imunoallergy, Luz Saúde, Hospital da Luz, Av. Lusíada 100, 1500-650 Lisbon, Portugal

**Keywords:** Pathogenesis, Eye diseases

## Abstract

To determine the association between hyperglycemia, glycated albumin (GlyA) and retinopathy of prematurity (ROP). Prospective study of all infants under ROP screening from March 2017 to July 2019. All demographic, clinical and laboratory data were collected. Glucose was measured at birth and every 8 h for the first week and serum GlyA was evaluated at birth, 1st, 2nd and 4th weeks after birth. Reference range for GlyA was obtained. Univariate logistic regression was used to examine risk factors for ROP followed by multivariate regression. A total of 152 infants were included in the study. Median gestational age was 30 weeks and median birth weight 1240 g. Thirty-three infants (21.7%) had ROP. Hyperglycemia was present in 24 (72.7%) infants diagnosed with any ROP versus 6 (0.05%) in those without ROP. Median GlyA at birth, 1st, 2nd and 4th and respective reference ranges were 8.50% (6.00–12.65), 8.20% (5.32–11.67), 8.00% (5.32–10.00) and 7.90% (5.30–9.00) respectively. After multivariate logistic regression, hyperglycemia but not GlyA, remained a significant risk factor for ROP overpowering the other recognized risk factors (Exp (B) 28.062, 95% CI for Exp(B) 7.881–99.924 p < 0.001). In our cohort, hyperglycemia but not GlyA, remained a significant risk factor for ROP overpowering the other recognized risk factors.

## Introduction

Retinopathy of prematurity (ROP) is a neovascular disorder of the immature retina and a leading cause of preventable blindness worldwide^1,2^. Neonatal hyperglycemia is a common problem in extremely preterm infants that has been linked to increased mortality and morbidity^3,4^. Several studies have also reported an association between hyperglycemia and ROP^5–17^.

Glycation of various proteins is known to occur at higher rate in individuals with hyperglycemia^18,19^. Albumin is more sensitive to glycation than other proteins, mainly because of its high concentration and long half-life and also due to the large number of lysine and arginine residues that may be involved in the formation of early and advanced glycation products^19^. Similarly to HbA1c, glycated albumin (GlyA) can be used as a clinical marker for ROP. GlyA is especially useful in newborns, as high levels of fetal hemoglobin affect HbA1c measurements and reflects plasma glucose levels over a shorter period due to the shorter half-life of serum albumin when compared to erythrocytes^20,21^.

The objective of this study was to determine the association between hyperglycemia, GlyA and ROP.

## Methods

### Study population

This was a prospective cohort study of all preterm infants who underwent ROP screening from March 2017 to July 2019, in two neonatal intensive care units from two institutions in Portugal. The study was approved by the Ethical Review Boards of Hospital São Francisco Xavier—Centro Hospitalar de Lisboa Ocidental and Hospital Beatriz Ângelo, as well as by the National Data Protection Authority (CNPD—Comissão Nacional de Proteção de Dados) and the Ethics Committee of the Nova Medical School. It was carried out in compliance with the principles of the Declaration of Helsinki, in its latest version (Brazil, 2003).

Following the current Portuguese screening guidelines, all infants with gestational age (GA) ≤ 32 weeks, birth weight (BW) ≤ 1.500 g, or being at higher risk of ROP determined by a neonatologist are eligible for ROP screening and were included in the study^22^. Infants without a known ROP outcome, and those with any ocular diseases apart from ROP were excluded.

### Data collection

For ROP screening, an initial fundus examination was performed at 32 weeks of postmenstrual age (PMA) or at 4 weeks of chronological age, whichever was later. The diagnosis of ROP and indication of treatment for ROP followed the International Classification of ROP Revisited^23^ and the Early Treatment for ROP Study^24^, respectively. The term severe ROP included both “type 1 ROP” and “type 2 ROP” both defined by the criteria of the Early Treatment for ROP study^24^.

Demographic and ophthalmologic data, medications, laboratory results, diagnosis, procedures and maternal history were collected.

Following the neonatal units’ protocol, glucose concentration was measured at birth and daily, every 8 h, during the first week using a point-of-care glucometer. For any glucose reading of ≤ 50 or ≥ 150 mg/dl, a serum sample was sent to the laboratory for confirmation. Infants were stratified into two groups: a hyperglycemia group (when plasma glucose concentrations exceeded > 150 mg/dl at least once for the first 7 days after birth) and an euglycaemia group (50–150 mg/dl). Hypoglycemia was defined as blood glucose ≤ 50 mg/dl.

Serum GlyA was evaluated at birth, 1 week, 2 and 4 weeks after birth using the leftover blood collected for purposes other than the study. Serum GlyA was determined by an enzymatic method using albumin-specific protease, ketoamine oxidase and albumin assay reagent (QuantILab Glycated Albumin, Instrumentation Laboratory SpA—A Werfen Company, Milan, Italy). GlyA was hydrolyzed by an albumin-specific protease, and then oxidized by ketoamine oxidase to produce hydrogen peroxide, which was measured quantitatively. Serum GlyA levels were calculated as the percentage of GlyA relative to total albumin, which was measured in the same serum sample using the bromocresol purple method^25,26^.

### Statistical methods

Univariate logistic regression was used to examine risk factors for ROP. In this analysis, we evaluated GA, BW, sex, small for GA status, gestational diabetes, hyperglycemia, exposure to insulin, GlyA (birth, 1st, 2nd and 4th week after birth), days before first discharge, hyaline membrane disease, bronchopulmonary dysplasia, need of oxygen > 28 days, days of mechanical ventilation, days of oxygen supplementation, thrombocytopenia (≤ 50,000 platelets per microliter), thrombocytosis (≥ 500 × 10^3^/l), postnatal steroids (dexamethasone), early red cell transfusion (first 10 days after birth), total red cell transfusions, anemia (< 11 g/dl) during the first week after birth, persistent *ductus arteriosus*, peri and intraventricular hemorrhage, diuretics (furosemide), days of phototherapy, early and late onset sepsis, necrotizing enterocolitis (NEC) and absence of maternal milk.

Univariate analysis was followed by multivariate regression (backward conditional) using variables that were significant on univariate and clinically important. Each multivariable logistic regression analysis included a maximum of four independent variables always with the same dependent value, which was presence, or absence of ROP. All multivariable regression analyses were performed using a backward conditional method. We considered a p < 0.05 statistically significant. Reference range for GlyA was obtained according to the CLSI EP28-A3C^27^. Data analysis was performed using the SPSSv23 (IBM, USA).

### Ethics approval and consent to participate

This research complies with the guidelines for human studies and was conducted ethically in accordance with the World Medical Association Declaration of Helsinki. Parents/guardians have given their written informed consent and the study protocol was approved by the relevant ethics committees (Ethical Review Board of Centro Hospitalar de Lisboa Ocidental reference number 172/CES—2015; Ethical Review Board of Hospital Beatriz Ângelo reference number 1944/2017_MJHNO; National Data Protection Authority (CNPD—Comissão Nacional de Protecção de Dados) reference number 9377/2016 and the Ethics Research Committee NMS|FCM-UNL (CEFCM) reference number 28/2016/CEFCM).

## Results

A total of 152 infants were included in the study. Median GA was 30 weeks (range 24–36 weeks) and the median BW was 1240 g (range 408–2670 g). Thirty-three infants (21.7%) developed ROP, 20 (13.2%) severe ROP and 6 (3.9%) Type 1 ROP (Tables 1, 2).

**Table 1:** Rate of ROP stratified by gestational age and birth weight.

	No ROP n (%)	Any ROP n (%)	Severe ROP n (%)	Type 1 ROP n (%)
**Gestational age (weeks)**				
24–25	2 (1.6)	5 (15.2)	5 (25.0)	4 (66.7)
26–27	8 (6.7)	10 (30.3)	6 (30.0)	1 (16.7)
28–29	23 (19.3)	12 (36.4)	6 (30.0)	1 (16.7)
30–32	70 (58.8)	6 (18.2)	3 (15.0)	0 (0.0)
> 32	16 (13.4)	0 (0.0)	0 (0.0)	0 (0.0)
**Birth weight (g)**				
< 750	5 (4.2)	13 (39.4)	9 (45.0)	3 (50.0)
750–1000	13 (10.9)	9 (27.3)	5 (25.0)	2 (33.3)
1001–1500	78 (65.5)	11 (33.3)	6 (30.0)	1 (16.7)
1501–1800	20 (16.8)	0 (0.0)	0 (0.0)	0 (0.0)
> 1801	3 (2.5)	0 (0.0)	0 (0.0)	0 (0.0)
Total	119	33	20	6

**Table 2:** Characteristics of the included infants.

n	Total cohort	No ROP	Any ROP	Severe ROP	Type 1 ROP
	152	119	33	20	6
Male, n (%)	78 (51.3)	65 (54.6)	13 (39.4)	9 (45.0)	2 (33.3)
Gestational age (weeks) (median, range)	30 (24–36)	31 (24–36)	28(24–32)	27 (24–30)	25 (24–29)
Birth weight (g) (median, range)	1240 (408–2670)	1335 (500–2670)	825 (408–1340)	770 (408–1340)	736 (589–110)
SGA, n (%)	37 (24.3)	27 (22.7)	10 (30.3)	7 (35.0)	2 (33.3)
Gestational diabetes, n (%)	18 (11.8)	14 (11.7)	4 (12.1)	2 (10)	0 (0.0)
Hyperglycemia, n (%)	30 (19.7)	6 (5.0)	24 (72.7)	15 (75.0)	4 (66.7)
Hyaline membrane disease* n (%)	132 (86.8)	101 (84.8)	31 (93.9)	18 (90.0)	6 (100.0)
Bronchopulmonary dysplasia, n (%)	43 (28.3)	20 (16.8)	23 (69.6)	15 (75.0)	6 (100.0)
Need of oxygen > 28 days, n (%)	47 (30.9)	22 (18.5)	25 (75.8)	17 (85.0)	6 (100.0)
Days of mechanical ventilation (median, range)	0 (0–86)	0 (0–86)	7 (0–82)	17 (0–60)	24 (0–60)
Days of oxygen supplementation (median, range)	10 (0–226)	8 (0–226)	46 (1–221)	72 (1–221)	89 (41–221)
Thrombocytopenia** n (%)	47 (30.9)	33 (27.7)	14 (42.4)	10 (50.0)	3 (50.0)
Prenatal steroids for pulmonary maturation n (%)	139 (91.4)	108 (90.8)	31 (93.9)	19 (95.0)	5 (83.3)
Postnatal steroids n (%)	7 (4.6)	3 (2.5)	4 (12.1)	3 (15)	3 (50.0)
Early red cell transfusion^+^ n (%)	27 (17.8)	10 (8.4)	17 (51.5)	13 (65.0)	5 (83.3)
Total red cell transfusions (median, range)	0 (0–13)	0 (0–8)	2 (0–13)	4 (0–13)	7 (0–9)
Anemia first week of life^++^ n (%)	25 (16.4)	11 (9.2)	14 (42.4)	10 (50.0)	3 (50.0)
Persistent ductus arteriosus n (%)	22 (16.4)	14 (11.7)	8 (24.2)	5 (25.0)	2 (33.3)
Peri and intraventricular hemorrhage^+++^ n (%)	46 (30.3)	29 (24.4)	17 (51.5)	14 (70.0)	5 (83.3)
Furosemide n (%)	38 (25.0)	20 (16.8)	18 (54.5)	13 (65.0)	4 (66.7)
Days of phototherapy (median, range)	3 (0–15)	3 (0–15)	4 (0–9)	4 (0–9)	4 (2–7)
Early onset sepsis n (%)	55 (36.2)	38 (31.9)	17 (51.5)	13 (65.0)	4 (66.7)
Late onset sepsis n (%)	60 (39.5)	37 (31.1)	23 (69.6)	15 (75.0)	3 (50.0)
NEC n (%)	8 (5.3)	2 (1.7)	6 (18.2)	5 (25.0)	1 (16.7)

*NEC* necrotizing enterocolitis.

*Grade 1–4.

** ≤ 50 000 platelets per microlite.

^+^First 10 days after birth.

^++^Hemoglobin < 11 g/dl.

^+++^Grade I–IV.

Hyperglycemia was present in 24 (72.7%) infants diagnosed with ROP versus 6 (5.0%) without ROP. Median glycaemia was 95 mg/dl in infants with ROP and 85 mg/dl in those without (p < 0.001). The median number of occurrences of hyperglycemia in the first week was 2 (range 1–6). No infant required insulin. Postnatal steroids were all prescribed after the first month after birth.

An interim analysis of GlyA was made for the first 94 infants included in the study (Table 3). Having more than half of the cohort analyzed and since GlyA was not found to be a significant risk factor (p = 0.629, p = 0.408, p = 0.278, p = 0.064, at birth, 1 week, 2 week and 4 week, respectively), it was decided not to pursue with its analysis to all cohort.

**Table 3:** Summary of glycated albumin values (serum glycated albumin (GA) levels are calculated as the percentage of GA relative to total albumin).

	Glycated albumin (%)	All (n = 94)	No ROP (n = 74)	Any ROP (n = 20)
Birth	Median (%) (range)	8.50 (6.30–17.50)	8.50 (6.30–17.50)	8.50 (7.20–10.10)
1 week after birth	Median (%) (range)	8.20 (5.30–12.10)	8.20 (5.30–12.10)	8.10 (6.50–10.50)
2 weeks after birth	Median (%) (range)	8.00 (5.10–18.20)	7.90 (5.50–18.20)	8.30 (5.10–10.00)
4 weeks after birth	Median (%) (range)	7.90 (5.50–10.00)	7.90 (5.50–10.00)	7.75 (6.50–9.80)

GlyA values in infants that were not treated with postnatal corticosteroids permitted us to determine a reference range according to the CLSI EP28-A3C^27^ (Table 4). There was no statistical difference in GlyA values between infants that received prenatal corticoids *vs.* those who did not (p = 0.557, 0.153, p = 0.361, p = 0.831 at birth, 1 week, 2 week and 4 week, respectively).

**Table 4:** Reference range for glycated albumin in preterm infants (serum glycated albumin (GA) levels are calculated as the percentage of GA relative to total albumin).

	At birth	First week	Second week	Fourth week
Glycated albumin reference range (%)	6.000–12.650	5.325–11.675	5.325–10.000	5.300–9-000

Risk factors for ROP identified in univariate logistic regression were GA, BW, hyperglycemia, days before first discharge, hyaline membrane disease type 3, bronchopulmonary dysplasia, need of oxygen > 28 days, days of mechanical ventilation, days of oxygen supplementation, early red cell transfusion (first 10 days after birth), total red cell transfusions, anemia (< 11 g/dl) during the first week after birth, late onset sepsis, diuretics and NEC.

Risk factors identified after multivariate logistic regression included BW, hyperglycemia, need of oxygen > 28 days and NEC (Table 5).

**Table 5:** Risk factors for ROP after multivariate regression analyses.

	B	Exp(B)	95% CI for Exp(B)	*p*-value
Birth weight	− 0.004	0.996	0.993–0.998	< 0.001
Hyperglycemia	3.334	28.062	7.881–99.924	< 0.001
Oxygen > 28 days	2.399	11.012	3.132–38.716	< 0.001
Necrotizing enterocolitis	2.617	13.693	1.361–131.714	0.026

## Discussion

Despite recent advances in neonatal care, ROP remains a great source of morbidity among the very low weight infants^1^. While immaturity and supplemental oxygen are already known major risk factors, ROP is a multifactorial disease process with many associated risk factors^17^. Our aim was to determine the association between hyperglycemia, GlyA and ROP and further compare them with other known risk factors. After multivariate logistic regression, hyperglycemia but not GlyA, remained a significant risk factor for ROP overpowering the other recognized risk factors.

Hyperglycemia is a common metabolic disturbance affecting up to 80% of very low birth infants^4,28^. In preterm infants, it has been associated with low insulin like growth factor 1 (IGF-1) levels, which are essential for adequate retinal neovascularization^29^. Hyperglycemia is associated to oxidative stress and leads to increased resistance of vessel walls and changes in organ blood flow, thus exacerbating retinal hypoxia^30^.

Garg et al., in a retrospective case–control study of 16 infants were the first to associate increased glucose levels in the initial month after birth to ROP^17^. Since then, 10 retrospective studies^5–7,10–16^ and one prospective study^8^ reported a significant association between hyperglycemia and ROP. Lee et al.^31^ in a retrospective study of 24,548 infants with hyperglycemia, (blood glucose > 180 mg/dl), concluded that hyperglycemia alone was not associated with severe ROP. However, blood glucose > 150 mg/dl and insulin use were associated with severe ROP. In our study, hyperglycemia was defined as blood glucose > 150 mg/dl but no infant needed insulin.

In 2019, a study by Jagla et al.^32^ reported that higher glycemic variability during the first week after birth was associated with treatment requiring ROP. However, this association was lost on logistic regression. This loss might be due to real-time clinical intervention correcting the glucose level and limiting its consequences or due to the inability to detect differences due to relatively small sample size (n = 152). The same author assessed the association between glycemic variability in the 1st week after birth and type 1 ROP in a case–control study of 40 premature infants. After multiple regression, risk of type 1 ROP was only found to be associated with duration of oxygen exposure and higher glycemic variability^33^. More recently, Cakir et al.^29^ in a cohort of 117 infants, reported that mean plasma glucose levels > 135.32 mg/dl during first week after birth were associated with lower postnatal IGF-1 levels and increased risk of ROP. These findings were replicated in a neonatal hyperglycemia oxygen-induced retinopathy mouse model, demonstrating that decreased insulin signaling suppressed liver production, lowered serum IGF-1 levels and increased retinal neovascularization. In our cohort, the median glycaemia was 95 mg/dl in infants with ROP and 85 mg/dl in those without, supporting that the difference in median glycaemia between these two groups is not large. Nevertheless, a trend for higher glycaemia does seem to exist in infants that develop ROP. Furthermore, and in line with the studies mentioned above, we also found that the presence of any event related to hyperglycemia was more frequent in infants diagnosed with ROP versus in those without (72.7% vs. 5.0%) even after multivariate analysis.

Due to the proposed association between hyperglycemia and ROP, the authors tried to uncover if GlyA’s values differed between infants with and without ROP and if so, if it would serve as clinical marker for ROP. Analogously to HbA1c, GlyA is one of the clinical markers for glycemic control and has been proven to be useful in the care of patients with neonatal diabetes mellitus^18,34,35^. Its main advantage over the widely used HbA1c, is that GlyA is not affected by hemoglobin’s metabolism or its variants^35^. Neonatal blood contains a high proportion of fetal hemoglobin (HbF)^20,36^. Additionally, preterm infants commonly undergo red blood cell transfusions which would also impair true evaluation of glycemic control with HbA1c. GlyA has a high specificity as it reflects glycation products of a single protein, is not affected by the concentration of serum protein because it is expressed as a ratio to albumin, nor is affected by other proteins^34^.

In the particular case of infants at risk for ROP, GlyA has the advantage of reflecting plasma glucose levels over a shorter period. Because the half-life of serum albumin (14 days) is shorter than that of erythrocytes, it reflects the status of glycemic control changes for 2–4 weeks^20,35^. Moreover, GlyA reflects the fluctuation of plasma glucose as well as the mean plasma glucose^34^, thus having the potential of reflecting the glycemic control during the first phase of ROP. Due to the exploratory nature of our study the authors believed that the chosen sampling intervals (birth, 1 week, 2 and 4 weeks) would maximize the ability to detect relevant changes. Koga et al.^36^ have previously studied the GlyA levels in the umbilical cord blood of 5 neonates at birth. Serum median GlyA level was 9.4 ± 1.1% (slightly lower than the lower limit of normal controls for adults). Suzuki et al.^20^ tried to establish the reference intervals for GlyA in 58 healthy full-term newborn infants. Age-dependent reference values (95% CI) for GlyA in infants were between 4.9–9.4% at 4–7 days, 5.5–10.1% 7–14 at 7–14 days and 6.2–10.8% at 2–4 weeks. In our cohort, the obtained median GlyA values in the 4 time points were similar to those reported by Suzuki et al. albeit with a greater range of values.

Because this was a proof of concept an interim analysis of GlyA was made halfway thru the study and it was found that GlyA alone was not a significant risk factor and therefore it was decided not to continue this analysis. GlyA can be altered in patients with albumin metabolism disorders^20^. None of our infants had nephrotic syndrome, or altered thyroid function, however most of them received prenatal corticoids. Two studies have reported the effect of antenatally administered corticosteroids: one study did not show an effect on whole body amino acid metabolism^37^, whereas in another, infants that received prenatal corticosteroids for lung maturation tended to have a 37% increase in albumin synthesis, although not statistically significant^38^. In our study there was no statistical difference in GlyA values between infants that received prenatal corticoids vs. those who did not.

Yudkoff et al. reported that the relatively low serum albumin concentrations, typical of premature infants, appear to be referable to more rapid turnover of a small plasma pool rather than a diminution in the rate of albumin synthesis^39^. It is possible that the rapid turnover did not allow for sufficient glycation. Other possible reason for the lack of difference between GlyA in ROP and non-ROP infants might be due to the insufficient duration of hyperglycemia. Both data from the Continuous Glucose Monitoring in Very Preterm Infants^40^ and from 2 other similar studies^28,32^ monitoring the first week after birth reported that the time spent in the predefined hyperglycemia was low (2.3% to 10.3%^28,32,40^). One hypothesis is that the occurrence rather than the duration of hyperglycemia might be associated with ROP.

Although not significant as a risk factor, with this study, we provide a reference range for GlyA at four different time points in premature infants that can be useful to assess the accurate glycemic status in these babies. Neonatal diabetes may affect gestational age and so a percentage of patients could present prematurely, or certain mutations may result in premature birth^41^. In a cohort of 750 patients with neonatal diabetes, 16% of patients with a genetic diagnosis were born prematurely^41^. There are several limitations of our study, namely the small cohort, the use of ROP rather than treatment requiring ROP (due to limited sample size) and the inclusion of only two institutions from the same geographical area. We also recorded hyperglycemic events rather than its duration, due to the inability to continuously monitor the glucose level in the entire cohort.

In conclusion, after multivariate analysis, the classic known risk factors—BW, need of oxygen > 28 days and NEC—continued to be associated with ROP. Nevertheless, hyperglycemia remained a significant risk factor for ROP, overpowering the other recognized risk factors. Although not linked to an increased risk of ROP we have provided for the first time a reference range for GlyA in premature infants, that can be used in future studies as a tool to evaluate glycemic control. Future studies should aim to prospectively investigate the effects of glucose control, preferably with continuous monitoring, in larger cohorts of premature infants, to better ascertain whether blood glucose level/variability is a specific risk factor for ROP or rather an indirect marker reflecting the severity of the newborns’ morbidity, and in turn conferring a higher risk for ROP.

## Data Availability

The datasets used and/or analyzed during the current study are available from the corresponding author upon reasonable request.
